# Blinded by PRISMA: Are Systematic Reviewers Focusing on PRISMA and Ignoring Other Guidelines?

**DOI:** 10.1371/journal.pone.0096407

**Published:** 2014-05-01

**Authors:** Padhraig S. Fleming, Despina Koletsi, Nikolaos Pandis

**Affiliations:** 1 Oral Growth and Development, Barts and The London School of Medicine and Dentistry, Institute of Dentistry, Queen Mary University of London, London, England; 2 Department of Orthodontics, University of Athens, Athens, Greece; 3 Department of Orthodontics, University of Bern, Bern, Switzerland; Toronto Western Hospital, Canada

## Abstract

**Background:**

PRISMA guidelines have been developed to improve the reporting of systematic reviews (SRs). Other reporting guidelines and techniques to assess methodological quality of SRs have been developed. We aimed to assess the frequency of the use of reporting and other guidelines in SRs to assess whether PRISMA is being used inappropriately as a substitute for other relevant guidelines.

**Methods:**

Web of Knowledge was searched to identify articles citing the PRISMA guidelines over a 12-month period. The use of reporting guidelines (including PRISMA and MOOSE) and tools for assessing methodological quality (including QUADAS) was assessed. Factors associated with appropriate use of guidelines including review type, field of publication and involvement of a methodologist were investigated.

**Results:**

Over the 12-month period, 701 SRs were identified. MOOSE guidelines were cited in just 17% of epidemiologic reviews; QUADAS or QUADAS-2 was referred to in just 40% of diagnostic SRs. In the multivariable analysis, medical field of publication and methodologist involvement (OR = 1.97, 95% CI: 1.37, 2.83) were significant predictors of appropriate use of guidelines. Inclusion of a meta-analysis resulted in 73% higher odds of appropriate usage of systematic review guidelines (OR = 1.73, 95% CI: 1.22, 2.35).

**Conclusions:**

Usage of SR reporting guidelines and tools for assessment of methodological quality other than PRISMA may be under-utilized with negative implications both for the reporting and methodological quality of systematic reviews.

## Introduction

Systematic reviews (SRs) are “prepared using a systematic approach to minimizing biases and random errors which is documented in a materials and methods section” [1] and have become established as a linchpin of evidence-based practice, influencing clinical practice and informing health policy. It is, therefore, important that SRs adhere to rigorous methodology and are clear and unbiased. While interventional SRs are prominent in the assessment of the comparative effectiveness of healthcare procedures, other reviews including epidemiologic reviews, reviews of diagnostic tests, qualitative reviews and individual patient meta-analysis are commonplace and increasingly influential [2]. There is, therefore, a similar onus on clear and accurate reporting of these types of SR.

A plethora of reporting and methodological guidelines have been developed to assist in the reporting and conduct of all aspects of research, including SRs [3] and randomized controlled trials (CONSORT [4] and its extensions) and observational studies (e.g. STROBE [5]). These guidelines have had a positive impact on the reporting of research, although further work and awareness is required to elevate the standard of reporting to required levels [6]–[8]. Appropriate reporting of meta-analyses was initially outlined in the QUOROM statement [9]. This was subsequently updated to encompass systematic reviews generally. However, guidelines and adjuncts tailored specifically to each type of systematic review exist. For example, MOOSE guidelines [10] guide the reporting and conduct of epidemiologic reviews, AMSTAR is accepted as a valid tool for assessing the methodological quality of interventional SRs [11], while QUADAS [12] and latterly QUADAS-2 [13] have been developed to aid methodological assessment in diagnostic reviews.

Inadequate reporting risks shrouding the conduct of systematic reviews impairing potential judgements in relation to the emphasis that should be placed upon their results and conclusions. It is, therefore, important that these guidelines are considered and applied where necessary to optimise both the conduct and reporting of all types of SR. The aims of the present research were to assess the frequency of correct use of appropriate guidelines in SRs and to highlight factors associated with inappropriate use or omission of relevant guidelines.

## Methods

Articles citing the PRISMA guidelines were searched from January 1^st^ to December 31^st^ 2012, using the Web of Knowledge database (http://apps.webofknowledge.com) by one of the authors (PSF). The first step consisted of a search for PRISMA publications on the PRISMA website (http://prisma-statement.org). The ‘Create citation report’ tool on the Web of Knowledge database was accessed to identify articles citing one of these six PRISMA guideline articles. SRs only were to be included in the research with other article types including narrative reviews, meta-epidemiologic studies, letters, editorials and other studies excluded from the analysis.

Two researchers (PSF and DK) screened the titles and abstracts of all retrieved references. Electronic versions of potentially eligible reviews were retrieved and analyzed by the authors to assess eligibility. Review articles without a specific methodology section were excluded. Two reviewers independently extracted data from eligible reviews using piloted forms. Calibration was initially undertaken on 10 articles and disagreements were resolved through consensus or by discussion with a third reviewer (NP).

Data were obtained on the type of systematic review, medical field of publication, number of authors, involvement of a methodologist or statistician in the paper, specific PRISMA article cited, reason for the citation, and whether meta-analysis was undertaken. The involvement of a methodologist was gauged from affiliations or qualifications reported and information in the review methodology section.

Descriptive statistics were performed to categorize the number of SRs identified within each medical field and to relate reporting to the remaining study characteristics. Pearson chi-squared test and Fisher’s exact test were used to determine the association between appropriate PRISMA citation and trial characteristics including medical specialty, number of authors, methodologist involvement, specific PRISMA statement article cited, reason for citation, type of SR, and implementation of meta-analysis or otherwise. Univariable and multivariable logistic regression modeling was used to determine the association between appropriate PRISMA statement citation and predictor variables including medical field of research, number of authors, methodologist involvement, cited article and whether meta-analysis was undertaken. The adequacy of the model was assessed using the Hosmer-Lemeshow test. The level of statistical significance for all tests was pre-specified at 0.05. All statistical analyses were conducted with statistical software (Stata 12.1, Stata Corp, College Station, TX, USA).

## Results

**Figure 1:**
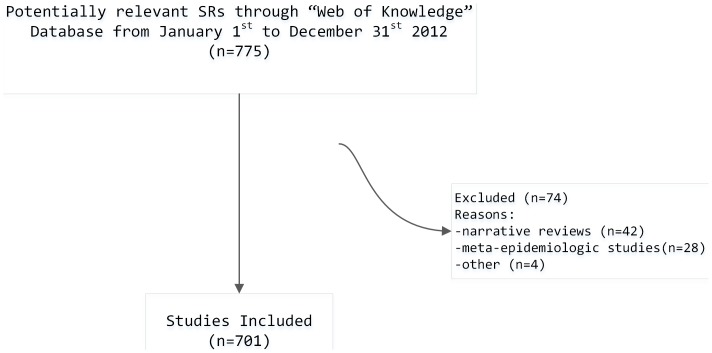
Flow diagram of article retrieval.

Seven hundred and one SRs were identified pertaining to 10 medical fields (Figure 1, Table 1). The highest number of SRs was published in General Medical (n = 121), Public Health (n = 103) and Cardiovascular (n = 93) fields. The majority of studies were authored by more than 6 researchers without the involvement of a methodologist and included meta-analysis. Most were interventional SRs, citing PRISMA as a guideline for reporting reasons, while the most frequently cited article was that of Moher *et al*., (2009) published in PLoS Medicine. The guideline most frequently omitted within the subset of SRs citing only PRISMA [3] was MOOSE [9] (Table 2), with 236/284 (83%) of epidemiologic reviews citing the latter guidelines, in isolation. Similarly, in SRs of diagnostic tests 23/57 (40%) cited PRISMA without referring to either QUADAS or QUADAS-2, the remainder cited PRISMA^3^ in isolation.

**Table 1:** Characteristics of the included SRs (n = 701).

	Appropriate PRISMA citation		Citation of PRISMA without complementary citation of other relevant guideline			Total		p-value
	No.	%	No.	%		No.	%	
**Medical Field**								
*Public Health*	44	10	59	22		103	15	<0.001*
*Psychiatry/Neurology*	28	6	35	13		63	9	
*Oncology*	47	11	41	16		88	13	
*Other*	55	13	33	13		88	13	
*Obstetrics*	30	7	14	5		44	6	
*General Medicine*	85	19	36	14		121	17	
*General Surgery*	30	7	10	4		40	6	
*Cardiovascular*	74	17	19	7		93	13	
*Orthopedics/Physiotherapy*	28	6	10	4		38	5	
*Dentistry*	17	4	6	2		23	3	
**Number of authors**								
*1–3*	114	26	63	24		117	25	0.34*
*4–5*	152	35	82	31		234	33	
*≥6*	172	39	118	45		290	41	
**Methodologist Involvement**								
*No*	264	60	190	72		454	65	0.001*
*Yes*	174	40	73	28		247	35	
**Cited PRISMA article**								
*BMJ*	113	26	76	29	189		27	0.79^≠^
*JCE*	61	14	27	10	88		13	
*PLoS Med*	159	36	100	38	259		37	
*IJS*	18	4	9	3	27		4	
*Ann Intern Med*	83	19	50	19	133		19	
*Phys Ther*	4	1	1	0	5		1	
**Reason for citing PRISMA**								
*Reporting*	402	92	220	84	622		89	<0.01^≠^
*Flow diagram*	23	5	17	6	40		6	
*Illustrative*	3	1	6	2	9		1	
*Quality assessment*	1	0	6	2	7		1	
*Search strategy*	9	2	14	5	23		3	
**Type of SR**								
*Interventional*	356	81	4	2	360		51	<0.001^≠^
*Epidemiological*	48	11	236	90	284		41	
*Diagnostic*	34	8	23	9	57		8	
**Meta-analysis**								
*No*	126	29	116	44	242		35	<0.001*
*Yes*	312	71	147	56	459		65	
**Total**	**438**	**100**	**263**	**100**	**701**		**100**	

*Pearson chi^2^, ^≠^ Fisher’s exact test.

**Table 2:** Omitted guideline among the subset of SRs citing PRISMA, based on field of publication (n = 263).

Medical Field	Omitted guideline			Total
	MOOSE	QUADAS/QUADAS-2	AMSTAR	
	N (%)	N (%)	N (%)	N (%)
*Public Health*	55 (23)	4 (17)	0 (0)	59 (22)
*Psychiatry/Neurology*	32 (14)	2 (9)	1 (25)	35 (13)
*Oncology*	38 (16)	2 (9)	1 (25)	41 (16)
*Other*	29 (12)	4 (17)	0 (0)	33 (13)
*Obstetrics*	11 (5)	1 (4)	2 (50)	14 (5)
*General Medicine*	33 (14)	3 (13)	0 (0)	36 (14)
*General Surgery*	9 (4)	1 (4)	0 (0)	10 (4)
*Cardiovascular*	18 (7)	1 (4)	0 (0)	19 (7)
*Orthopedics/Physiotherapy*	7 (3)	3 (13)	0 (0)	10 (4)
*Dentistry*	4 (2)	2 (9)	0 (0)	6 (2)
**Total**	236 (100)	23 (100)	4 (100)	263 (100)

**Figure 2:**
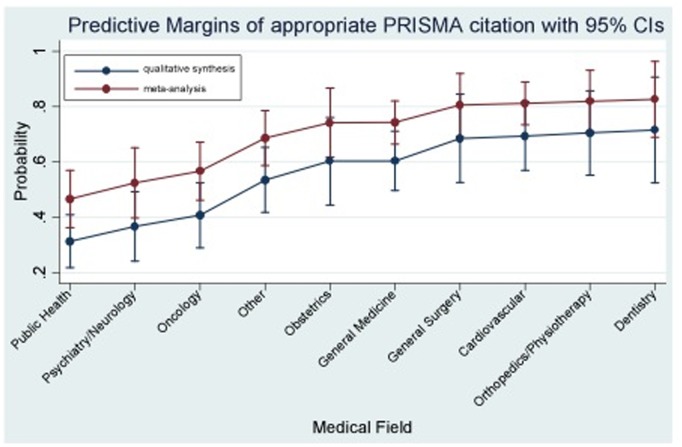
Predicted probabilities of appropriate PRISMA citation with 95% confidence intervals (CIs) derived from the adjusted model based on the medical field and implementation of a meta-analysis within the SR.

In the univariable analysis (Table 3), medical field of research was associated with appropriate PRISMA [3] citation (Other: OR = 2.23, 95% CI: 1.25, 4.0; Obstetrics: OR = 2.87, 95% CI: 1.36, 6.05; General Medicine: OR = 3.17, 95% CI: 1.82, 5.50; General Surgery: OR = 4.02, 95% CI: 1.78, 9.09; Cardiovascular: OR = 5.22, 95% CI: 2.76, 9.88; Orthopedics/Physiotherapy: OR = 3.75, 95% CI: 1.65, 8.53; Dentistry: OR = 3.80, 95% CI: 1.38, 10.42; compared to Public Health). Involvement of a methodologist and use of meta-analysis were also identified as significant predictors for the outcome. In the multivariable analysis (Table 3), medical field (Other: OR = 2.57, 95% CI: 1.41, 4.69; Obstetrics: 3.40, 95% CI: 1.58, 7.33; General Medicine: OR = 3.41, 95% CI: 1.93, 6.04; General Surgery: OR = 4.91, 95% CI: 2.12, 11.37; Cardiovascular: OR = 5.11, 95% CI: 2.63, 9.91; Orthopedics/Physiotherapy: OR = 5.40, 95% CI: 2.30, 12.64; Dentistry: OR = 5.68; 95% CI: 2.01, 16.02; compared to Public Health) and methodologist involvement (OR = 1.97, 95% CI: 1.37, 2.83; compared to non-involvement) remained significant predictors of appropriate PRISMA citation. There was also evidence that inclusion of a meta-analysis was associated with appropriate citation of SR guidelines (OR = 1.73, 95% CI: 1.22, 2.35). The predicted probabilities from the adjusted model for appropriate PRISMA citation are shown by medical field and inclusion of a meta-analysis or otherwise in Figure 2.

**Table 3:** Univariable and multivariable logistic regression derived ORs and 95% confidence intervals (CI) for appropriate PRISMA citation in the identified SRs (n = 701).

Predictor variables		Univariable analysis			Multivariable analysis		
Variable	Category	OR	95% CI	p-value	OR	95% CI	p-value
**Medical Field**	*Public Health*	Baseline (reference)					
	*Psychiatry/Neurology*	1.07	0.57, 2.02	0.83	1.28	0.66, 2.45	0.47
	*Oncology*	1.54	0.87, 2.73	0.14	1.52	0.84, 2.73	0.17
	*Other*	2.23	1.25, 4.00	<0.01	2.57	1.41, 4.69	<0.01
	*Obstetrics*	2.87	1.36, 6.05	<0.01	3.40	1.58, 7.33	<0.01
	*General Medicine*	3.17	1.82, 5.50	<0.001	3.41	1.93, 6.04	<0.001
	*General Surgery*	4.02	1.78, 9.09	0.001	4.91	2.12, 11.37	<0.001
	*Cardiovascular*	5.22	2.76, 9.88	<0.001	5.11	2.63, 9.91	<0.001
	*Orthopedics/Physiotherapy*	3.75	1.65, 8.53	<0.01	5.40	2.30, 12.64	<0.001
	*Dentistry*	3.80	1.38, 10.42	0.01	5.68	2.01, 16.02	0.001
**No authors**	*1–3*	1.24	0.84, 1.83	0.27			
	*4–5*	1.27	0.89, 1.81	0.19			
	*≥6*	Baseline (reference)					
**Methodologist Involvement**	*No*	Baseline (reference)					
	*Yes*	1.72	1.23, 2.39	0.001	1.97	1.37, 2.83	<0.001
**Cited article for PRISMA**	*BMJ*	Baseline (reference)					
	*JCE*	1.52	0.89, 2.60	0.13			
	*PLOS med*	1.06	0.72, 1.56	0.75			
	*IJS*	1.35	0.57, 3.15	0.50			
	*Ann Intern Med*	1.12	0.71, 1.76	0.64			
	*Phys Ther*	2.69	0.29, 24.54	0.38			
	*PLOS med (Liberati)*	1.35	0.24, 7.53	0.74			
**Meta-analysis**	*No*	Baseline (reference)					
	*Yes*	1.95	1.42, 2.69	<0.001	1.73	1.22, 2.45	<0.01

## Discussion

The requirement for high quality systematic reviews of healthcare interventions, epidemiological data and diagnostic information is clear as the emphasis on evidence-based decision-making continues. A corollary of this situation is for more complete and transparent reporting of the design and conduct of a SR, allowing stakeholders to reach informed decisions based on the validity of the findings. The PRISMA guidelines [3] were developed with the expressed aim of standardizing reporting and enhancing the clarity of reporting of SRs. While the emphasis of the guidelines changed from a focus on interventional research to a more generic approach from its precursor [9], the PRISMA [3] guidelines do not consider nuances of certain aspects of other review types. It is, therefore, important that its use is supplemented by reference to additional tailored reporting and quality assessment guidelines in respect of these review types. The findings from the present study, however, indicate that a significant percentage of epidemiologic and diagnostic reviews are published without reference to these complementary resources.

While there is significant overlap between MOOSE [10] and PRISMA [3], there are items unique to each with MOOSE [10] guidelines comprised of 35 items. In particular, MOOSE [10] incorporates very detailed instructions in respect of search strategy, including delineation of the qualifications of the searchers, use of hand-searching and approaches to dealing with unpublished and non-English literature, emphasizing the centrality of this aspect of the review to meta-analysis. The importance of the assessment of the potential for bias in primary studies is reinforced in both guidelines. However, greater emphasis is placed on the interpretation of the results of the review, specifically regarding possible alternate explanations for the observed findings in the MOOSE guidelines [10]. This distinction reflects the elevated susceptibility of observational research to both bias and confounding limiting the potential inferences and the degree to which the results of the review can be trusted and used to inform healthcare decisions [14]. The finding from the present study of 83% of epidemiologic reviews citing PRISMA [3] without referring to MOOSE [10] suggests that these reviews may lack complete reporting of these methodological aspects and failure to explore the reasons for the observed findings in sufficient detail.

Meta-analyses of diagnostic tests have become increasingly prominent in medicine and will potentially have an increasing role in healthcare as decision makers consult the evidence before implementing new diagnostic technologies. It is important that such analyses provide reliable results; this is contingent upon both the quality of conduct and the clarity of reporting. As with over review types, diagnostic systematic reviews are susceptible to shortcomings pertaining to conduct and reporting [15]–[17]. To mitigate potential weaknesses, quality assessment of the primary studies has become integral to diagnostic reviews; the QUADAS [12] and more recently QUADAS-2 [13] guidelines have facilitated this process. The present study, however, revealed that 40% of diagnostic reviews did not refer to either of these guidelines risking inadequate methodological comparison of constituent primary studies in diagnostic reviews.

Disentanglement of the conduct of SRs from reporting is more complex than is the case with primary studies, as significant overlap exists between the two. Nevertheless, PRISMA guidelines [3] were developed with the intention of improving the reporting of reviews; the AMSTAR guidelines [11] have emerged as a valid tool to assess the conduct of SRs. While meta-epidemiologic studies were omitted from the analysis in the present cross-sectional study, it was noted that 14% of these also failed to refer to AMSTAR guidelines [11] during methodological assessment of SRs suggesting an over-reliance on PRISMA [3] in this further respect. It is, therefore, important that greater awareness of these guidelines arises.

The over-reliance on PRISMA guidelines [3] in respect of reporting of SRs reflects widespread awareness and endorsement of these guidelines. This is certainly a positive development, which has led to enhanced reporting of SRs [6], although further work is required [7], [8]. However, while a plethora of reporting guidelines are available in a prominent on-line resource [18], lack of awareness of alternatives and complementary guidelines is potentially problematic. This situation may relate to widespread espousal and endorsement of reporting guidelines including PRISMA [3] and CONSORT [4] by biomedical journals, while other guidelines, including MOOSE [10] and QUADAS [12], have not been treated similarly. In recent times efforts have been made to improve compliance with PRISMA guidelines using active editorial involvement [6]; these efforts have not been replicated in respect of other guidelines, including those pertaining to observational research. It is likely, therefore, that over-reliance on PRISMA will continue, unless similar efforts are made to improve awareness of complementary guidelines. It is, therefore, important that emphasis on the reporting of not just interventional reviews but also epidemiologic, diagnostic and other systematic reviews is enhanced. This development will be facilitated by raising awareness of other guidelines, such as MOOSE [10] and QUADAS-2 [13] and encouraging active editorial input to enhance reporting of all review types.

Potential limitations of this study included the relatively short time period assessed; however, a large number of eligible studies from diverse areas of medicine were included. Nevertheless, temporal changes in reporting characteristics will not be identified. In addition, given that the focus of the research was to assess the usage of PRISMA guidelines specifically, it is likely that epidemiologic and diagnostic reviews that cite more specific guidelines in isolation, without referring to PRISMA, exist.

## Conclusions

There is evidence that significant awareness of PRISMA guidelines exist. However, usage of other accepted reporting guidelines and tools for assessment of methodological quality have less prominence and may be under-utilized with negative connotations on both reporting and methodological quality of systematic reviews.
